# Inter-provincial variation in older home care clients and their pathways: a population-based retrospective cohort study in Canada

**DOI:** 10.1186/s12877-023-04097-5

**Published:** 2023-06-26

**Authors:** Lori Mitchell, Jeffrey Poss, Martha MacDonald, Rosanne Burke, Janice M. Keefe

**Affiliations:** 1 Shared Health Manitoba, Winnipeg, MB Canada; 2 School of Public Health Sciences, Faculty of Health, University of Waterloo, Waterloo, ON Canada; 3 Saint Mary’s University, Halifax, NS Canada; 4 Nova Scotia Centre On Aging, Mount Saint Vincent University, Halifax, NS Canada; 5 Department of Family Studies and Gerontology and Director, Nova Scotia Centre On Aging, Mount Saint Vincent University, Halifax, NS Canada

**Keywords:** Canadian Home Care, Client pathways, Client outcomes, interRAI

## Abstract

**Background:**

In Canada, publicly-funded home care programs enable older adults to remain and be cared for in their home for as long as possible but they often differ in types of services offered, and the way services are delivered. This paper examines whether these differing approaches to care shape the pathway that home care clients will take. Older adult client pathways refer to trajectories within, and out of, the home care system (e.g., improvement, long term care (LTC) placement, death).

**Methods:**

A retrospective analysis of home care assessment data (RAI-HC was linked with health administrative data, long-term care admissions and vital statistics in Nova Scotia Health (NSH) and Winnipeg Regional Health Authority (WRHA). The study cohort consists of clients age 60 + years, admitted to home care between January 1, 2011 to December 31, 2013 and up to four years from baseline. Differences in home care service use, client characteristics and their pathways were tested across the two jurisdictions overall, and among the four discharge streams within jurisdictions using t-tests and chi-square tests of significance.

**Results:**

NS and WHRA clients were similar in age, sex, and marital status. NS clients had higher levels of need (ADL, cognitive impairment, CHESS) at base line and were more likely discharged to LTC (43% compared to 38% in WRHA). Caregiver distress was a factor correlated with being discharged to LTC. While a third remained as home care clients after 4 years; more than half were no longer in the community – either discharged to LTC placement or death. Such discharges occurred on average at around two years, a relatively short time period.

**Conclusions:**

By following older clients over 4 years, we provide enhanced evidence of client pathways, the characteristics that influence these paths, as well as the length of time to the outcomes. This evidence is central to identification of clients at risk in the community and aids in planning for future home care servicing needs that will allow more older adults to remain living in the community.

**Supplementary Information:**

The online version contains supplementary material available at 10.1186/s12877-023-04097-5.

## Background

In Canada, provincial and territorial governments are shifting their reliance on facility-based care to care in the home for older adults [1, 2] with home care services, in particular, increasing in importance as a result [3–6]. This shift serves the preference of older adults to remain and be cared for in their home for as long as possible [7–10]. The benefits of home care services for older adults can include improved functioning and quality of life [11–13], delayed admission to a long-term care facility, or decreased use of other health care services [14, 15]. This shift to community care over facility-based care for older adults also aligns with an important consideration for governments, namely cost of care, as home care services are generally less costly forms of service for older adults [13, 15–17]. In 2017 the Government of Canada confirmed an investment of $6 billion over ten years to improve home and community care services in Canada in response to Canadians’ increased demands for this service and to reduce reliance on costlier acute care services [2].

Where populations are aging [18, 19], a growing number of older adults require home care to support their goal of remaining in their homes for as long as possible [17]. Appropriate investment in home care to meet this demand requires a broad understanding of home care service delivery and client pathways once on the service. However, home care studies often do not fully explore the pathways of home care clients since they are focused on singular trajectories of clients, such as long-term care (LTC) [17] or assisted living placement [20]. Little research addresses the multiple trajectories that clients will take while on a home care caseload. In addition to moving to facility-based care, some clients will end home care services either because their health improved and they no longer need the service, or they opted for alternative delivery than the public system. Other clients will die while on the home care caseload or soon after transitioning to facility-based care. Even others will remain on home care services over a projected period of time.

Another gap in published research is the limited timeframe to observe client pathways. For example, the risk for hospitalization or accessing emergency department care only followed home care clients for a one-year period [21]. A recent study identified that the majority of long-stay home care clients in Canada are still active on home care after 2 years (71%) [17], which suggests that longer follow up periods are required to examine the pathways of clients through the home care system. Analysis of home care clients’ trajectories are needed over an extended period of time. Previous approaches in study methods narrow the ability to fully explore the complexity of client trajectories in home care and patterns in service delivery associated with each path.

In Canada, each province or territory sets their own eligibility criteria for public home care services [22, 23] which results in differing client characteristics and care needs when older adults start services [24, 25]. Comparative analyses can provide evidence of the influence that provincial/territorial policies and contexts have on client populations, yet there is limited information on potential variation among jurisdictions in their approaches to home care service for older adults and how that may affect the client pathway through the home care system. For example, jurisdictions differ in the services covered, hours of care allocated, fees, and the provision of services (public provision or contracted to non-profit or for-profit agencies).

This study addresses some of the gaps in previous research by examining large cohorts of older home care clients in two Canadian jurisdictions and discerning all pathways and outcomes over an extended period of time. We will identify and examine individual and home care service factors related to an older client’s pathway in a population-based cohort of home care clients after admission to publicly funded home care. Our objectives are to: a) examine and describe the older client population at admission to home care and over time – up to four years after admission; b) review patterns of home care service after admission and the pathways and outcomes of home care clients; and c) compare and contrast clients’ clinical status and trajectory of home care between two Canadian jurisdictions. This study is part of a larger research program that aims to use a mix of research methods to enhance understanding of the pathways of older adults with chronic and long-term conditions through home care [26].

## Methods

### Study design and settings

This retrospective population-based cohort study used multiple linked clinical and administrative datasets to examine clinical status and public home care services for a cohort of older home care clients. Each client in the cohort was followed from baseline to up to four years to examine their pathway through home care.

This study was conducted in two Canadian jurisdictions – the greater metropolitan area of the city of Winnipeg (serviced by the Winnipeg Regional Health Authority (WRHA), the largest health authority in the province of Manitoba) and the province of Nova Scotia (serviced by Nova Scotia Health (NSH), Nova Scotia’s single health authority). Both jurisdictions have public home care programs but differing approaches to service delivery. In Nova Scotia, home care services are provided by private agencies that are contracted through NSH while in the WRHA/Manitoba most home care services are delivered by public employees of the health authority’s Home Care Program. In both jurisdictions, public home care is comprised of non-professional personal support and homemaking services, and professional health services, namely nursing services [26].

### Study cohort

Home care services can be provided to meet a variety of needs, such as short-term recovery care from an acute condition, support for rehabilitation, longer-term care to maintain individuals with frailty and chronic conditions in the community, or end of life care [4]. The focus of this research is on long-stay home care clients (receive home care for 60 days or longer) since this study aims to enhance understanding of home care client pathways of older adults with chronic and long-term conditions. The study cohort consisted of all community-dwelling, long-stay home care clients in the public WRHA and NSH Home Care Programs, inclusive of goals of care for rehabilitation, community maintenance, or palliative purposes. Short-term or nursing-only clients were excluded from the study.

The clients were admitted and had an initial clinical assessment for home care between January 1, 2011 and December 31, 2013. The cohort was age 60 or older at the time of their admission assessment and were on home care for a minimum of 60 days. If a client had multiple home care episodes, i.e., multiple admissions in the 2011 to 2013 period, the first episode in the period that was for a minimum of 60 days was selected for baseline. The clients in the cohort were required to have at least two clinical assessments completed, an admission and at least one subsequent assessment, so that any changes in clinical status in the client could be examined. We also required that at a minimum some non-professional services were received, but we allowed up to 120 days for this to be observed due to the possibility of wait times for home care service early in an episode of care.

### Jurisdiction differences

Home care policies and entitlements vary across Canada and this is no different in NSH and WRHA. Nursing services in both jurisdictions are insured services. As part of the NSH program, clients are means-tested and the cost of the home support services (S12.45 per hour) are based on a sliding household income scale. For example, a single person with net income of between $26,166 to $41,165 would pay $12.45 per hour for home support to a maximum monthly client fee of $125 (a couple would be exempt from payment at this income level). Among the highest household income over $71,000, the maximum monthly client fee would be $622 [27]. WHRA clients are not income-tested therefore, access to home support care program is based solely on need. Another difference is the way services are delivered. In Winnipeg, home care and support services are provided in-house by WRHA employees [28]; in NS the Health Authority contracts these services out to private agencies to deliver across the Province. There are 5 for-profit and 19 not-for-profit home care agencies across the province and during the time of the study clients were on wait lists to obtain home care supports [29]. Despite these differences, in both jurisdictions a client is assessed by the public home care programs’ case manager to determine eligibility for service and the type and amount of service required. Therefore, assessment, care plans and service allocation are the responsibility of the two public home care programs involved in this study. Similarly, in both jurisdictions, the same public home care case managers assess for eligibility for long term care placement. As a result, two key features that can influence a client’s pathway throughout a home care episode – the need for and amount of home care service, and the need to transition to long term care—are in the public home care domain.

### Data sources

Analytic data sets were constructed independently for the WRHA and NSH jurisdictions, using encrypted data sources that were substantively the same, including:

- Resident Assessment Instrument for Home Care (RAI-HC), the clinical assessment that the home care programs in both jurisdictions utilize. The RAI-HC is a standardized, comprehensive assessment designed for use with adult and non-palliative home care clients expected to be on service for at least 60 days. It forms the basis for the Canadian Institute for Health Information (CIHI) national reporting system for home care [30], has acceptable reliability and validity [31, 32], and has been used extensively for both public reporting [25, 33] and for research [34–37]. Long-stay adult home care clients in both jurisdictions receive an initial assessment on referral to home care, and are expected to be re-assessed annually, or earlier in the case of a significant clinical change. As noted, the clients in the cohort had a minimum of two RAI-HC assessments to allow examination of change over time. The second RAI-HC assessment could be administered up to four years after the initial assessment, and all RAI-HC assessments up to four years were considered in the analysis.
- The Discharge Abstract Database [38] is the CIHI standard for acute care hospitalizations and is used here to inform both absences from home while in hospital, and trajectories through hospital to discharge (death or to LTC).
- Emergency Department visits were extracted from repositories maintained by each jurisdiction and are used to inform deaths that are recorded there.
- Vital statistics are maintained by each province, used here to inform dates of death.
- Data on amount, type, and timing of home care services were as follows: In the WRHA these were informed from individual staff visits which record the date, duration, and discipline providing the visit. NS data were in the form of *client service plans* that cover a specific period of time, from which hours of care by week can be derived. In both jurisdictions, services for nursing as well as home support (for assistance with activities of daily living (ADL) such as dressing or bathing and other non-professional services) were available.
- Informing discharge from home care differed somewhat between the two jurisdictions. In the WRHA, the RAI-HC database contained explicit records with discharge dates and reasons and were supplemented by a LTC placement file that recorded dates of entry. In NS, service plan records recorded dates of long-term care home placement, however home care discharge was mostly inferred by examining dates of death, long-term care placement, and cessation of a home care services plan.

For each jurisdiction, only records from the above sources for the eligible cohort and years were made available, based on the study sampling framework.

### Measures

Descriptive characteristics were drawn from a client’s first and last RAI-HC assessments. Selected measures and several embedded scales from the RAI-HC clinical data informed client health status indicators at baseline and over time: information about informal caregiving (hours and co-residing with a caregiver) and caregiver distress (a caregiver is unable to continue, or the primary caregiver expresses depression, anger, or distress); the ADL hierarchy scale expresses impairments with activities of daily living and is scored from 0 (independent) to 6 (completely dependent) [39]; the Cognitive Performance Scale is also scored from 0 (cognitively intact) to 6 (very severe impairment) with values of 3 or greater corresponding to MMSE scores of approximately 15 or lower [40]; CHESS (Changes in Health, End-stage, Signs, Symptoms) provides a measure of health instability, with values of 2 or greater having moderate or higher levels [41]; the DRS (Depression Rating Scale) uses 7 depressive symptoms to create a 0 to 14 point scale with values of 3 or greater associated with significant depression [42]; and MAPLe (Method for Assigning Priority Levels) scores of high or very high denote significantly elevated likelihood among home care clients for nursing home placement [43].

The geographical location (urban/rural) of the NS clients was reviewed and, because no important differences emerged, NS clients were treated as one group.

One of four discharge pathways was assigned hierarchically, up to four years after the first assessment: 1) client died while home care was active, or during a hospitalization that began when home care was active; 2) client was placed in a long-term care home and did not return to the community to receive home support service again within the four year period; 3) client was receiving home support service during the last three months of the four year period; and 4) all other clients, including those who transferred out of the jurisdiction or otherwise left home care and did not return to service within the 4 year period.

The measure of home support and nursing hours per week was based on periods up to 90 days after a RAI-HC assessment; specifically, the first and last visits in those periods were used as end-points for aggregating the sum of home support hours and the span of time (converted to weeks). We looked for evidence of discontinuity in home support service, based on a period of 30 days or longer prior to discharge, at any point after home support service began, in which no home support service could be observed, and there were no in-hospital days in that period.

### Statistical analysis

Statistical differences between continuous measures used T-tests, and chi-square tests were used for dichotomous or nominal outcomes. Differences were tested across the two jurisdictions overall, and among the four discharge groups or pathways within jurisdictions, but not directly for the matching jurisdiction and discharge combinations.

It is important to make the distinction that, although assignment to the four discharge pathways is based on the destination after leaving home care, our data could inform those who subsequently died who were discharged from home care to long-term care, or elsewhere. At three-month intervals starting at the baseline RAI-HC assessment, cases were assigned to one of four mutually exclusive states: receiving home care, in LTC, deceased (including those who subsequently died after discharge from home care), or alive and not receiving home care.

We considered the potential issue of comparing two populations that were geographically distinct with different make-ups. The WRHA is a concentrated urban area and excludes other urban areas as well as all rural and remote areas of Manitoba. Our Nova Scotia population represents the entire province, including three possibly distinct segments: Halifax as a large urban area, other smaller urban areas, and rural areas. We examined the extent to which home care in these three Nova Scotia segments was distinct, either in client characteristics, services, or discharge pathways, using the client’s Forward Sortation Area (first 3 digits of the Postal Code), matched to a list of Halifax metropolitan area codes for the first group, and using the second digit zero convention to assign rural clients [44].

Analysis was performed in SAS 9.4 (WRHA) and SAS Enterprise Guide 7.12 (NS).

## Results

A total of 10,601 home care clients were included in this study. There were approximately 5,300 cases in both the WRHA and NS cohorts, summarized in Table 1. Within the NS population, 25% resided in the Halifax area, 33% in other urban areas, and 43% in rural areas. Client characteristics were generally similar among the three segments (detailed results not shown), with Halifax clients more likely than rural clients to have high levels of instrumental ADL difficulties (80% vs 60%) and responsive behaviours (17% vs 10%). A difference in healthcare aide time was also noted, with nearly 7 h per day for Halifax clients compared to 5.4 h for rural clients, but this was mainly explained by client case mix differences, including the two aspects mentioned. A review of the discharge pathways did not reveal a significant difference between NS urban and rural clients. Similar proportions and length of time to LTC placement was also observed. Based on these results, the aggregation of all NS clients into one group was considered appropriate for this examination.

**Table 1:** Descriptive Characteristics at Initial Assessment, by Province and Discharge Pathway (%^a^)

	**WRHA**					**Nova Scotia**				
	died	To LTC	still on service after 4 years	all other discharges	**All**	died	To LTC	still on service after 4 years	all other discharges	**All**
N (% of provincial sample)	989 (18.7)	1,713 (32.5)	2,029 (38.4)	547 (10.4)	**5,278 (100.0)**	977 (18.4)	2,306 (43.3)	1,681 (31.6)	359 (6.7)	**5,323 (100.0)**
female	56.2	65.0	72.6	70.2	**66.8**	58.9	71.4	73.9	68.2	**69.7**
mean age (std dev)	81.9 (8.5)	83.7 (7.4)	80. (8.7)	78.6 (8.7)	**81.4 (8.5)**	81.6 (8.9)	82.6 (7.8)	78.2 (8.2)	78.6 (8.8)	**80.7 (8.4)**
over 85	41.3	48.7	31.3	26.3	**38.3**	40.3	40.9	21.5	28.5	**33.8**
live alone at referral	48.0	54.8	59.0	52.4	**54.9**	41.5	48.1	53.9	52.2	**49.0**
married	38.2	35.9	33.2	38.9	**35.6**	37.8	33.7	33.4	33.7	**34.3**
co-resides with informal caregiver	49.3	44.9	38.8	47.6	**43.7**	54.0	47.1	39.7	42.4	**45.7**
no informal caregiver	1.2	1.0	1.8	0.9	**1.3**	1.8	1.3	3.7	2.9	**2.3**
caregiver distress	19.0	24.6	14.3	18.6	**19.0**	24.1	28.3	15.8	18.9	**22.9**
primary caregiver child/child-in-law	51.6	55.5	56.5	52.0	**54.8**	48.7	55.9	52.8	49.7	**53.2**
primary caregiver is spouse	30.2	26.9	23.1	27.5	**26.1**	30.1	25.3	24.1	25.5	**25.8**
mean informal hrs in 7 days (std dev)	14.1 (16.1)	15.7 (19.7)	10.3 (13.6)	10.8 (12.9)	**12.8 (16.4)**	28.8 (34.2)	29.7 (36.1)	19.1 (27.2)	21.4 (29.1)	**25.7 (33.1)**
ADL hierarchy 1 or greater	28.3	28.5	19.9	17.6	**24.0**	39.5	40.2	22.8	32.6	**34.0**
CPS 3 or greater	5.5	10.9	2.2	3.7	**5.8**	9.9	15.7	4.1	5.7	**10.3**
CHESS 2 +	39.3	33.7	31.1	28.4	**33.2**	54.0	52.9	41.8	41.0	**48.8**
DRS 3 +	8.6	9.0	8.6	7.8	**8.6**	18.0	24.5	16.0	17.7	**20.2**
daily pain	52.4	44.5	62.6	51.9	**53.7**	50.9	49.8	62.5	62.0	**54.8**
MAPLe high or very high	9.2	52.0	18.3	31.1	**32.6**	40.7	57.6	25.1	31.3	**42.5**
urinary incont. at least 2 × per week	23.4	27.9	19.4	17.9	**22.7**	24.4	28.6	22.9	22.6	**25.6**
any aggressive behaviour	2.5	6.0	1.3	2.0	**3.1**	11.4	17.6	5.6	6.5	**11.9**
1 or more falls last 90 days	36.6	39.7	35.4	30.4	**36.5**	48.0	49.2	42.1	41.9	**46.2**
4 or more diagnoses	46.9	40.2	40.4	39.8	**41.5**	60.3	56.2	56.7	53.3	**56.9**
Alzheimer’s/related dementia	4.2	34.0	7.4	14.0	**18.0**	19.5	37.0	9.4	14.4	**23.5**
stroke	16.2	14.9	15.2	19.0	**15.7**	116.1	13.7	13.6	14.1	**14.1**
heart failure	19.4	9.7	9.2	10.5	**11.4**	12.6	8.5	7.6	7.3	**8.9**
cancer	15.8	8.0	9.4	9.8	**10.2**	15.2	10.2	9.8	11.4	**11.1**
psychiatric diagnosis	12.3	14.1	15.1	16.5	**14.4**	11.9	16.5	16.0	17.7	**15.6**
COPD	21.4	13.1	14.9	14.4	**15.5**	26.9	15.9	22.1	21.7	**20.3**
diabetes	26.1	17.3	21.8	22.0	**21.2**	29.7	24.9	31.8	27.5	**28.1**
arthritis	46.7	48.5	55.3	47.2	**50.6**	58.0	60.8	68.7	59.5	**62.7**

^a^unless otherwise indicated

Most characteristics in Table 1 showed statistical differences between the two jurisdictions, with the exceptions of proportion that were married, with daily pain, or with diagnoses of cancer or psychiatric conditions. A table showing statistical differences is provided in Additional file 1. Generally, NS clients had higher levels of need at these initial assessments, including receiving help for activities of daily living, and having cognitive impairment. Especially notable in the NS cohort were higher levels of health instability, depressive symptoms, aggressive/responsive behaviours, recent falls, and chronic conditions including dementia, COPD, diabetes, and arthritis. WRHA clients were more likely to live in a congregate care residence and to live alone. Hours of informal (unpaid) assistance were much higher in NS, as were markers for caregiver distress.

Considering discharge pathways, similar proportions were discharged deceased in both jurisdictions (almost 1 in 5), but notably more clients in NS were discharged to LTC and more clients in the WRHA were still supported by home care 4 years later. Most characteristics showed similar differences by pathway across both jurisdictions, for example cognitive impairment and dementia were most prevalent at the initial assessment among those who were discharged to LTC, and least prevalent among those still on home care after 4 years. Only a few characteristics showed little difference across the discharge groups: depressive symptoms in the WRHA, psychiatric diagnosis in the WRHA, and stroke in both jurisdictions. As expected, cancer was most prevalent among those discharged deceased, and caregiver distress highest among those that went to LTC. Those discharged to LTC were older, especially in the WRHA. Among those still on service at 4 years, higher prevalence of arthritis and daily pain were notable.

Table 2 presents additional episode, re-assessment, and services information. Clients who were discharged to LTC did so on average about 5 months earlier in the NS cohort, compared to WRHA cases. Assessment frequency shows consistent patterns across jurisdictions, with those going to LTC having the most frequent clinical re-assessment where it may be driven both by changing needs as well as administrative requirements that RAI-HC assessments be current for LTC placement to go forward. Clients remaining on service were assessed less than once per year on average, and the “other” discharges are notable in that assessment was more frequent in the WRHA, suggesting either some clinical or administrative differences in this group, compared to those in NS.

**Table 2:** Assessment, Home Support, and Nursing Services, by Province and Discharge Pathway

	**WRHA**					**Nova Scotia**				
	died	LTC	still on service after 4 years	all other discharges	**All**	died	LTC	still on service after 4 years	all other discharges	**All**
*N*	*989*	*1,713*	*2,029*	*547*	***5,278***	*977*	*2,306*	*1,681*	*359*	***5,323***
mean years to discharge/end of observation	2.2	2.2	4.0	2.3	**2.9**	2.2	1.7	4.0	3.1	**2.6**
Mean # of assessments per 12 months’ time	1.24	1.46	0.92	1.31	**1.13**	1.21	1.78	0.86	0.88	**1.18**
Home support hrs per week, at baseline^a^	4.66	5.25	3.93	3.99	**4.50**	6.42	6.71	4.69	5.00	**5.90**
Home support hrs per week, last 90 days observed^b^	7.92	9.51	5.41	4.81	**7.15**	11.24	11.04	7.29	7.16	**9.63**
*% increase baseline to end*	*36%*	*34%*	*35%*	*30%*	***35%***	*27%*	*25%*	*33%*	*29%*	***28%***
Any nursing, first 90 days	28%	18%	17%	17%	**19%**	39%	30%	27%	31%	**31%**
mean total hours (among those with any)	17.6	24.0	16.5	20.4	**19.4**	32.4	33.3	23.4	30.3	**29.8**
Proportion of these hours by RN	60%	57%	60%	65%	**59%**	36%	22%	32%	27%	**28%**
Any nursing, last 90 days	46%	25%	22%	20%	**27%**	61%	36%	36%	21%	**39%**
mean total hours (among those with any)	19.9	18.1	21.5	16.9	**19.4**	46.1	39.2	18.4	39.5	**33.9**
Proportion of these hours by RN	72%	53%	58%	53%	**61%**	42%	16%	25%	44%	**28%**

^a^in the 90 days after the first home support visit

^b^in the 90 days prior to the last home support visit observed in the 4-year period

Home support service averages show that NS had higher levels of planned service, in keeping with the generally higher needs. Rank order of home support intensity was consistent in the baseline 90-day period: highest intensity clients were among those eventually discharged to LTC, then those dying, with the lowest observed among those remaining on service. During the last 90 days, relative increases in home support levels were higher in the WRHA, overall. In the period after the client’s initial assessment, more clients in NS received one or more visits from a nurse, and their total volume of nursing service was higher. However, there was a significant difference in the proportion of nursing time provided by an RN, where it was around 60% in the WRHA compared to about 30% in NS. Nursing visit likelihood was highest among those who eventually were discharged deceased, both in the initial period, and especially in the period prior to discharge.

Similar to Table 1 and characteristics at initial assessment, Table 3 shows the values or proportions of client characteristics at the last RAI-HC assessment, and identifies where significant changes in client characteristics have occurred between clients’ initial (baseline) and last clinical assessment. Two related informal care items, the client co-residing with an informal caregiver, and the client having a primary caregiver who was a spouse, show a consistent decline in the proportion of clients with these characteristics over time, regardless of the clients’ discharge pathway. The proportion receiving informal support from an adult child/child-in-law increased among WRHA clients who remained on service, but not in NS. Caregiver distress increased overall but differed by discharge group, with WRHA cases remaining on service showing a significant decline, while NS clients remaining on service showed no significant change. In both jurisdictions the overall increase in caregiver distress was largely due to an increase in caregiver distress among clients who were discharged to LTC.

**Table 3:** Descriptive characteristics by province and discharge pathway at last assessment; changes since initial assessment*

	**WRHA**					**Nova Scotia**				
	died	To LTC	still on service after 4 years	all other discharges	***All***	died	To LTC	still on service after 4 years	all other discharges	***All***
N (% of provincial sample)	989 (18.7)	1,713 (32.5)	2,029 (38.4)	547 (10.4)	***5,278 (100)***	977 (18.4)	2,306 (43.3)	1,681 (31.6)	359 (6.7)	***5,323 (100)***
mean days between initial and last assessments (std dev)	593 (339)	619 (351)	1,193 (224)	636 (383)	***835 (419)***	596 (337)	584 (379)	1,178 (237)	794 (378)	***788 (428)***
co-resides with informal caregiver	46.6	^↓^39.5	^↓^33.1	^↓^40.0	^↓^***38.5***	52.5	^↓^40.2	36.2	42.2	^↓^***41.5***
no informal caregiver	1.0	1.1	1.8	0.9	***1.3***	1.5	1.3	^↓^2.5	2.7	***1.8***
caregiver distress	19.9	^↑^37.5	^↓^11.7	15.9	^↑^***22.1***	^↑^30.8	^↑^46.5	14.8	17.2	^↑^***31.7***
primary caregiver child/child-in-law	54.0	57.9	^↑^61.0	55.2	^↑^***58.1***	52.8	61.0	56.7	54.2	***57.6***
primary caregiver is spouse	27.6	24.9	^↓^18.8	24.0	^↓^***23.0***	27.5	^↓^21.7	^↓^19.9	22.8	^↓^***22.3***
mean informal hrs in 7 days (std dev)	^↑^16.4 (19.6)	^↑^19.5 (25.4)	11.1 (15.2)	11.6 (14.9)	^↑^***14.9 (20.2)***	^↑^30.2 (34.9)	^↑^28.9 (37.9)	19.5 (27.3)	20.6 (27.5)	^↑^***25.6 (34.0)***
ADL hierarchy 1 or greater	^↑^48.5	^↑^67.4	^↑^30.0	^↑^22.4	^↑^***44.8***	^↑^63.1	^↑^81.7	^↑^29.9	^↑^39.7	^↑^***59.3***
CPS 3 or greater	^↑^12.5	^↑^35.6	^↑^6.2	^↑^8.5	^↑^***17.1***	^↑^24.2	^↑^44.9	^↑^9.7	^↑^14.1	^↑^***28.0***
CHESS 2 +	43.4	^↑^47.4	^↓^23.0	^↓^18.7	***34.3***	^↑^59.9	^↑^65.1	^↓^34.7	37.1	^↑^***52.7***
DRS 3 +	10.6	^↑^17.2	7.3	8.2	^↑^***11.2***	^↑^26.3	^↑^34.5	18.2	21.7	^↑^***27.1***
daily pain	53.6	45.5	^↓^57.2	48.8	***51.8***	53.4	49.3	60.7	57.3	***54.1***
MAPLe high or very high	^↑^39.7	^↑^67.3	^↑^26.4	^↑^37.1	^↑^***43.3***	^↑^54.0	^↑^73.7	^↑^33.7	^↑^43.1	^↑^***55.6***
urinary incont. at least 2 × per week	^↑^36.0	^↑^51.8	^↑^32.1	^↑^24.5	^↑^***38.4***	^↑^38.7	^↑^49.4	^↑^32.4	^↑^29.7	^↑^***40.7***
any aggressive behaviour	^↑^6.5	^↑^16.3	^↑^3.2	^↑^4.1	^↑^***8.1***	^↑^19.7	^↑^32.5	^↑^10.6	^↑^13.0	^↑^***21.9***
1 or more falls	32.8	41.4	^↓^21.6	^↓^23.3	^↓^***30.3***	44.6	48.7	^↓^34.2	35.0	^↓^***42.5***
4 or more diagnoses	^↑^62.6	^↑^59.1	^↑^56.9	^↑^53.5	^↑^***58.3***	^↑^73.2	^↑^73.3	^↑^69.3	^↑^67.2	^↑^***71.6***
Alzheimer’s/related dementia	^↑^20.8	^↑^54.4	^↑^14.2	^↑^23.3	^↑^***29.4***	^↑^28.2	^↑^56.4	^↑^15.2	^↑^20.3	^↑^***35.8***
stroke	19.5	^↑^19.1	^↑^18.7	21.1	^↑^***19.2***	^↑^19.8	^↑^18.3	^↑^16.2	17.2	^↑^***17.9***
heart failure	^↑^27.2	^↑^14.2	^↑^14.9	13.0	^↑^***16.8***	^↑^18.1	^↑^11.5	^↑^9.7	10.3	^↑^***12.1***
cancer	^↑^21.1	9.7	10.7	11.9	^↑^***12.5***	^↑^20.8	^↑^12.6	10.6	14.7	^↑^***13.7***
psychiatric diagnosis	15.0	^↑^19.8	^↑^19.8	^↑^21.3	^↑^***19.1***	^↑^16.5	^↑^23.2	^↑^22.3	22.3	^↑^***21.6***
COPD	^↑^26.3	^↑^16.3	^↑^19.3	17.6	^↑^***19.5***	^↑^31.5	^↑^20.5	^↑^26.7	26.1	^↑^***24.9***
diabetes	27.5	19.0	23.8	24.7	^↑^***23.1***	32.1	26.5	32.7	33.0	^↑^***30.0***
arthritis	^↑^53.0	^↑^55.5	^↑^64.0	^↑^53.5	^↑^***58.0***	^↑^64.4	^↑^67.0	^↑^76.8	^↑^66.7	^↑^***69.4***

^*^↑ denotes, compared to initial assessment (in Table 1), a statistically significant (*p* < .05) *increase* in the proportion or mean value

↓ denotes, compared to initial assessment (in Table 1), a statistically significant (*p* < .05) *decrease* in the proportion or mean value

other cells were not significantly different from the baseline measures

Impairment in functional characteristics mostly increased over time, including physical dependency, cognitive impairment, urinary incontinence, aggressive behaviour, and a measure of overall risk of long-term care placement. Depressive symptoms tended to increase but did not show significant change among those remaining on service. Health instability (the CHESS scale) was lower, compared to baseline, among those remaining on service. In contrast, tendency to fall declined overall in both the WRHA and NS, most notably in those who remained on home care. Disease diagnoses also all tended to become more prevalent, with Alzheimer’s or a related dementia being the most likely new diagnosis, increasing from 18 to 29% in the WRHA, and from 24 to 36% in NS.

**Fig. 1:**
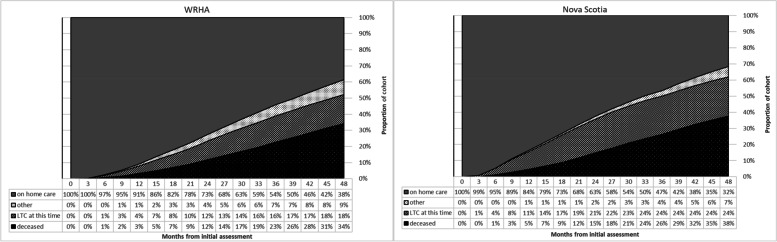
Area plot of cohort states over time by Province

Figure 1 shows clients’ states over time – whether they were still receiving home care, in LTC, deceased (including those who subsequently died after discharge from home care), or alive and not receiving home care, at 3-month intervals. The curves differ in that the NS cohort enters LTC much earlier, with 11% there within the first year, compared to 4% in the WRHA. A slightly higher proportion of the NS cohort was deceased at the end of 48 months (38%) compared to WRHA clients (34%); the proportion of clients becoming deceased starts to increase slightly more in Nova Scotia close to the 2-year mark. Note that the deceased category includes those discharged deceased while on active home care service, as well as those who died after discharge from home care (either through LTC, or some other discharge reason). But as noted in Table 1, 18.7% of WRHA clients and 18.4% of NS clients died while on home care. Therefore, a larger proportion of NS clients became deceased mainly while in LTC due to the greater proportion of clients discharged from home care to that location.

Additional files 1, 2 and 3 provide statistical levels resulting from comparisons in these data. Additional file 1 is a companion to Table 1; it gives *p*-values comparing the four discharge pathways in each cohort, and also comparing WRHA and NS cohorts overall. Not included are test values for the many additional pair-wise tests within and across the jurisdictions. Additional file 2 is a companion to Table 2; it provides similarly structured *p*-values for reassessment and services. Additional file 3 is a companion to Table 3; it gives the baseline and last assessment comparison *p*-values, by discharge pathway as well as overall, for the descriptive items in Table 3.

## Discussion

Given older adults’ desire to remain living in the community and ‘age in place’ [17] this research is providing valuable insight into the characteristics of older clients and their trajectories and outcomes once they are admitted to a public home care program. The majority of long-stay older clients can expect to remain in the community for about two years after home care admission. After that period of time discharges from home care increase in frequency. Client characteristics at admission and changes in client status figure prominently in a client’s pathway.

At home care admission, the clients in this study presented with characteristics similarly found in other studies of older home care clients – predominantly female, some level of ADL impairment more prevalent than moderate or greater cognitive impairment, and high prevalence of multiple chronic conditions [7, 15, 22, 45]. While half of this study’s clients lived alone at time of home care referral, nearly all of the clients had a family/friend caregiver present, as has previously been noted for home care clients in other studies [7, 46, 47].

However, client characteristics differed significantly when the two cohorts were examined individually, with NS clients presenting at admission to home care with significantly greater care and service requirements than the WRHA clients, entering public home care when their needs and impairment were at higher levels. Client characteristics at admission, whether lighter or heavier in need, are generally due to a jurisdiction’s eligibility criteria for services [47, 48]. Also access to home care programs may be curtailed because of insufficient services to meet the demand. NS contracts out the delivery of home support services to private agencies while WRHA uses internal health authority staff. In Nova Scotia where private agencies are contracted to deliver services, the relationship between having to wait for home support services and high care needs is unclear. However, it is possible that waitlists contributed to the deterioration of the client’s functional capacity and can partially explain the higher rates of discharge to LTC among the NS cohort after the first year on home care.

Another gap in knowledge is the ways in which the characteristics of older adult home care clients impact access to home care services [5]. Women access home care services earlier than men in their trajectories and have less medical intense needs [15]. Differences in client characteristics could also point to other access issues, such as when a person is eligible for care but cannot afford any payment for service that may be applied. As previously outlined, nursing services are provided to WRHA and NSH home care clients at no cost [28, 49, 50], but for home support services, Nova Scotians pay a fee for services based on their net household income up to a monthly maximum [27] while Manitobans do not. As a consequence, potential clients in Nova Scotia may delay care until a point in time when their needs are greatest, as the Canadian Foundation for Healthcare Improvement [51] found in its 2014 report. Conversely, in the WRHA’s Home Care program there is no income testing or fees for home support and nursing services [28, 50], which does support clients accessing services while at a lighter care need status.

The user fee structure in NS should be examined to ensure the appropriateness and fairness of fees for clients and sustainability of the services moving forward [52]. Differences in cost of home care services between jurisdictions may contribute to unmet needs among home care clients [4]. However, this partially contrasts with studies that have found that home care use was high even among clients that reported financial difficulty or of low socioeconomic status [4, 53].

This study reviewed the paths of home care clients over a longer time period than generally reviewed in previous home care research to more fully explore the trajectories of older home care clients in the long term. As expected, client characteristics when admitted to home care and changes in need influenced the paths of clients and the length of time they remained on home care. In this community-dwelling older adult home care population, overall, over one-third (35.0%) still remained on home care after four years post-admission. However, a slightly higher proportion overall (37.9%) were admitted to LTC and an additional 18.5% died within 4 years. Once on home care, few of these older long-stay clients were discharged from home care for reasons other than LTC placement or death, which is expected when long-term home care services are provided more to support older clients nearing the end of their time in the community rather than restoring function.

While over a third of older clients remained in the community for four years after entering home care, overall, more than half of the combined older clientele in the study were no longer in the community after four years, due to LTC placement or death. Such discharges occurred on average at around two years, a relatively short time. Similar to previous Canadian research the majority of the clients were still on home care at two years post-admission [17], but the shift to a minority of clients still remaining in the community occurred around the 3-year mark. This longitudinal review provides new evidence about client pathways and the length of time to those outcomes.

Evidence of the influence of the provincial context was found in this study as length of stay results shifted when the two cohorts were examined individually. A larger proportion of the WRHA cohort were still on home care service after four years (38.4%, compared to 31.6% of the NS cohort), while the majority of the NS cohort were admitted to LTC within the four years (43.3%, compared to 32.5% of the WRHA cohort). Moreover, the NS clients admitted to LTC were discharged from home care on average five months earlier than WRHA clients admitted to LTC. The difference in client status between the two cohorts when admitted to home care primarily drove this finding, as similar characteristics in general were found to be associated with the clients’ discharge disposition, regardless of jurisdiction. Higher prevalence of cognitive impairment, dementia, older age, and caregiver distress was found among clients discharged to LTC, risk factors similarly found in previous LTC transition research [17, 54–56]. Cancer, heart failure, and COPD were most prevalent among clients who deceased during the four years, while clients with lower levels of cognitive and physical impairment at admission and over time were still on home care after four years, also consistent with previous research [55]. The results do indicate though that the point at which an older adult accesses home care will influence which path they will take, and when.

The longer study review period also provided greater insight into health trajectories of older clients. Impairment in functional characteristics and disease diagnoses increased in prevalence over time among all the clients, with few exceptions. These results aid in determining where home care programs can focus clinical and disease-specific skills and resources. Alzheimer’s or a related dementia was the most likely new disease diagnosis to emerge among the elderly clients within the four years, with similar increases in prevalence of 11% in WRHA and 12% in NS clients over time. The prevalence of dementia was highest at baseline and had the greatest increase in prevalence over time among the clients that were discharged to LTC in this study. This result complements a previous Canadian study that demonstrated a new diagnosis of dementia is associated with LTC placement for community-dwelling older adults within five years [55]. Home care strategies to maintain clients with dementia safely in the community would assist many older adults to age in place.

Two notable exceptions to increased prevalence of impairment over time in both the WRHA and NS clients were for falls and health instability (CHESS score). Prevalence of these two characteristics decreased the most for clients who remained on home care after four years. For that client group, the prevalence of falls decreased over the four years from 35.4% to 21.6% in the WRHA and from 42.1% to 34.2% in NS, based on the review of their initial and last assessments (Table 3). Similar reductions in prevalence of health instability (CHESS score 2 +) were also found for these clients. These results point to a decrease in health instability and frailty over time that may have allowed the clients to remain in the community. Individuals who remained on home care did have lower levels of impairment at admission. Similarly, this group of clients did not have as large an increase in the number with physical or cognitive disability over time, compared to the clients who went to LTC. Previous research has found that home care service can improve health status and decrease falls [11]. The potential benefits of home care in our cohort may be greater when clients are less compromised to start, being better able to participate in or benefit from services and other community supports. The available data and methodology in this study did not allow us to determine the extent home care approaches may have contributed to clinical improvement found among clients who remained in the community within the study’s timeframe. Our results point to this group in home care populations that could be reviewed further in future research to understand what factors most contributed to their clinical improvement and maintenance in the community so that beneficial approaches could be applied to all clients.

The discharge pathways for our home care clients were primarily driven by their characteristics, which is a sign of appropriately functioning needs-based home care services. Client clinical need was also found to be appropriately related to patterns in home care approaches and servicing in this study overall and within sites, with client characteristics and worsening of status influencing the amount of home care service received, the frequency at which clients were reassessed, and involvement of nursing care alongside home support service. The greater impairment and care need found among the NS clients was associated with higher amounts of home support services and allocation of nursing services compared to the generally lower need WRHA cohort. Patterns such as this also point to home care services in our study sites being appropriately allocated based on client clinical need.

Other factors can influence client pathways, such as system capacity (LTC bed supply, the maximum amount of home care service that can be provided) [54] health service policy (home care discharge criteria, eligibility for LTC placement, fee payments) [5, 22, 57–60]; poor transitions between hospital and home (leading to premature admission to LTC) [22] and caregiver distress [61–63]. Such factors may account for the differences we found between the two jurisdictions. During the timeframe of this study, 2011 to 2017, Nova Scotia experienced a considerable increase in the number of LTC beds in the province [52]. For example, between 2006 and 2015, Nova Scotia added over 1000 LTC beds and replaced another 898 beds [52], while no new LTC beds were opened in the WRHA between 2011 and 2017 [64]. The increase in NS LTC beds in a similar timeframe to this research may have had some impact on the difference in LTC admissions. However, establishing that causal relationship was beyond the scope of this research and any correlation between the increase in LTC beds in NS and the higher rate of LTC admission found there was not systematically measured and can only be implied. Even though there were jurisdictional differences in client status when admitted to home care, there was striking similarity between the patterns, paths and outcomes of clients in NS and the WRHA, despite their great geographic distance and independent home care program development in Canada. There were similar case management approaches, based on reassessment frequency results. And similar client characteristics were associated with the client’s disposition from, or continuation on home care in both cohorts.

The length of time analyses for both sites also pointed to an average of 2 years as an important marker for remaining in the community before discharge to LTC or becoming deceased. There lies the window of opportunity for home care to potentially change the characteristics of a client’s path, through effective approaches such as restorative care/therapy services [65–68], targeted service for individuals with cognitive impairment [7, 69–72], disease management and stabilization [73], and services and supports (e.g., primary care integration) [3, 74–76] to slow disease progression [77]. Access to home care at earlier stages of need may also be beneficial [15]. Starting on home care when at greater levels of need may limit the ability to make improvements in a client’s status and change their path, considering this study found that some clients have a fairly short timeframe on the service.

Caregiver distress is another factor that emerged in the study that can influence client pathways, with an increase in caregiver distress over time being an impetus for LTC admission. Caregiver distress increased over time and was particularly high for NS caregivers when the client was discharged to LTC or died. These results mirror Betini and colleagues’ finding, using an equivalent data source from Ontario, namely that caregiver distress (and relationship) influence LTC admission – even after the residents’ health and age are taken into account [62]. Supports for caregivers have been prioritized by the NS Government in the last decade. Future research could examine the potential effect of caregiver supports such as, financial payment of the Caregiver Benefit, the Supportive Care payment, increases to respite services and government financial support to not for profit adult day programs and organizations that support caregivers such as Caregivers Nova Scotia and the Alzheimer Society of NS.

This study has a number of strengths. It was able to examine the pathways of older home care clients from two distinct Canadian jurisdictions over a 4-year time period. The standardized assessment data in both sites provided a unique comparison of clients and their status over the extended period of time; it demonstrates the benefit of cross-national use of the same clinical assessment for home care clients. The use of multiple administrative databases further strengthened the research and allowed for an in-depth review of client pathways. The results highlight for policy-makers and home care service providers the benefit of comparative designs to assess how the provincial context and approaches to access and care impact older clients. Future research should continue to evaluate comparative client characteristics, service delivery, and outcomes across regions and jurisdictions.

The study also has some limitations. The sample is not necessarily representative of all home care service clients, nor of all individuals who are assessed with the RAI-HC, due to the cohort criteria (e.g., age 60 or older) and restrictions for these analyses. The WRHA cohort is only representative of a large urban cohort, which limits the generalizability of the results from that site. However, older clients from the entire province of Nova Scotia (i.e., urban and rural) were included in that cohort to provide enhanced representation. Moreover, NS home care service data were based on care plan data not the actual service amount provided; WRHA service data were based on actual amounts provided. As a result, the NS service data may be over-estimated since sometimes services cannot be provided as planned. Nonetheless, the patterns found for Nova Scotia from the service data should not be affected, as the service amounts are an accurate reflection of what a case manager assessed the client as needing and believed the system could provide. As noted in the results section, NS data were reviewed for any rural/urban differences before aggregating the data, and no differential patterns in client pathways (discharge dispositions) were observed to indicate that actual service amounts may differ in the province, e.g., if there were greater difficulty servicing clients as planned in rural areas, the result might be more clients going to LTC, and sooner in rural areas.

Another limitation is the inability of this study to assess the impact of the different approaches to service delivery in each jurisdiction. Are WHRA clients at an advantage of having their services delivered by the same health authority that assesses them in comparison to NS where these services are contracted out to local agencies? What impact do these approaches have on the flexibility of staff and the relationships built between formal home caregivers and home care clients? Analysis of in-depth interviews with home care clients and their caregivers in the home care pathways project’s qualitative stream revealed how building flexibility into policies, protocols and institutional practice can better support client-centred care and provide more choice in care pathways over time [78].

This study found that allocation of home support and home nursing services in both jurisdictions were appropriately provided based on clinical need. However, clients may have experienced a level of unmet need, clinically or in other areas, that could influence their trajectories, given the impact on clients’ health and well-being when needs are not supported [4, 79]. Measurement of unmet need was not included in this study and future research incorporating such a focus in home care client pathways research would be beneficial.

## Conclusion

There is growing demand for community care by older adults voicing their preferences as well as governments seeking to reduce hospital stays and LTC transitions. These actions have placed greater attention on home care for the older population and ensuring the service meets clients’ needs to accomplish these objectives. With a longer review period, this study provides enhanced evidence of not only the pathways of older clients and the characteristics that influence those paths, but the length of time to those outcomes. This study found that level of client dependency was significantly associated with clients’ outcomes and the jurisdictional comparison highlighted how the timing of access to home care is associated with length of time on service. This evidence is central to identification of clients at risk in the community and aids in planning for future home care servicing needs that will allow more older adults to remain living in the community. There is considerable opportunity to further review the pathways of older home care clients, enhanced by jurisdictional comparison, as Canada’s population continues to age.

## Supplementary Information

**Additional file 1. **Statistical Difference *p* values: Descriptive Characteristics at Initial Assessment, by Province and Discharge Pathway (Reference Table 1).

**Additional file 2.** Statistical Difference *p* values: Re-Assessment and Services, by Province and Discharge Pathway (Reference Table 2).

**Additional file 3.** Statistical Difference *p* values for Descriptive Characteristics by Province and Discharge Pathway: Last assessment compared to Initial Assessments (Reference Table 3).

## Data Availability

The data that support the findings of this study are available from Health Data Nova Scotia, Nova Scotia Health, Nova Scotia Department of Seniors and Long Term Care and the Winnipeg Health Authority but restrictions apply to the availability of these data, which were used under license specific data sharing agreements for the current study, and so are not publicly available. Data are however available from Health Data Nova Scotia, Nova Scotia Health, Nova Scotia Department of Seniors and Long Term Care and the Winnipeg Health Authority through the appropriate research and approval processes of each of the respective organizations.

## Abbreviations

WRHA: Winnipeg Regional Health Authority
NS: Nova Scotia
RAI-HC: Resident Assessment Instrument for Home Care
LTC: Long-Term Care
RN: Registered Nurse
LPN: Licensed Practical Nurse
ADL: Activities of Daily Living
CHESS: Changes in Health, End-Stage Disease, Signs and Symptoms
CPS: Cognitive Performance Scale
DRS: Depression Rating Scale
MAPLe: Method for Assigning Priority Levels
COPD: Chronic Obstructive Pulmonary Disease
