# Supplementary material for: Inter-provincial variation in older home care clients and their pathways: a population-based retrospective cohort study in Canada
Source: BMC Geriatr. 2023 Jun 26;23:389. doi: 10.1186/s12877-023-04097-5 (PMC10291815; doi:10.1186/s12877-023-04097-5)
Supplement: Supplementary file 1 — Additional file 1. Statistical Difference p values: Descriptive Characteristics at Initial Assessment, by Province and Discharge Pathway (Reference Table 1). [file 12877_2023_4097_MOESM1_ESM.docx]

Additional file 1: Statistical Difference p values: Descriptive Characteristics at Initial Assessment, by Province and Discharge Pathway (Reference Table 1)

| Characteristics from RAI-Home Care | across 4 discharge groups, WRHA | across 4 discharge groups, NS | between WRHA and NS "All" |
| --- | --- | --- | --- |
| % of jurisdiction sample | **n/a** | **n/a** | **<.0001** |
| female | **<.0001** | **<.0001** | **0.0016** |
| mean age | **<.0001** | **<.0001** | **0.0002** |
| over 85 | **<.0001** | **<.0001** | **<.0001** |
| co-resides with primary or secondary | **<.0001** | **<.0001** | **0.0376** |
| married | **<.0001** | **<.0001** | 0.1605 |
| live alone at referral | **<.0001** | **<.0001** | **<.0001** |
| no informal caregiver | 0.1131 | **<.0001** | **0.0003** |
| caregiver distress | **<.0001** | **<.0001** | **<.0001** |
| primary caregiver child/child-in-law | **0.0059** | **<.0001** | 0.0981 |
| primary caregiver spouse | **0.0004** | **0.0023** | 0.7242 |
| mean informal hrs in 7 days | **<.0001** | **<.0001** | **<.0001** |
| ADL hierarchy 1 or greater | **<.0001** | **<.0001** | **<.0001** |
| CPS 3 or greater | **<.0001** | **<.0001** | **<.0001** |
| CHESS 2+ | **<.0001** | **<.0001** | **<.0001** |
| DRS 3+ | 0.7888 | **<.0001** | **<.0001** |
| daily pain | **<.0001** | **<.0001** | 0.2393 |
| MAPLe high or very high | **<.0001** | **<.0001** | **<.0001** |
| urinary incontinence at least 2x per week | **<.0001** | **<.0001** | **0.0006** |
| any aggressive behaviour | **<.0001** | **<.0001** | **<.0001** |
| any wandering | **<.0001** | **<.0001** | **<.0001** |
| 1 or more falls last 90 days | **0.0002** | **<.0001** | **<.0001** |
| 4 or more diagnoses | **0.0014** | **<.0001** | **<.0001** |
| Alzheimer’s/related dementia | **<.0001** | **<.0001** | **<.0001** |
| stroke | 0.2234 | 0.1552 | **0.0209** |
| heart failure | **<.0001** | **<.0001** | **<.0001** |
| cancer | **<.0001** | **0.0001** | 0.1472 |
| psychiatric diagnosis | 0.1186 | **0.0049** | 0.0964 |
| COPD | **<.0001** | **<.0001** | **<.0001** |
| diabetes | **<.0001** | **<.0001** | **<.0001** |
| arthritis | **<.0001** | **<.0001** | **<.0001** |
