# Supplementary material for: Inter-provincial variation in older home care clients and their pathways: a population-based retrospective cohort study in Canada
Source: BMC Geriatr. 2023 Jun 26;23:389. doi: 10.1186/s12877-023-04097-5 (PMC10291815; doi:10.1186/s12877-023-04097-5)
Supplement: Supplementary file 2 — Additional file 2. Statistical Difference p values: Re-Assessment and Services, by Province and Discharge Pathway (Reference Table 2). [file 12877_2023_4097_MOESM2_ESM.docx]

Additional file 2: Statistical Difference p values: Re-Assessment and Services, by Province and Discharge Pathway (Table 2)

|  | across 4 discharge groups, WRHA | across 4 discharge groups, NS | between WRHA and NS "All" |
| --- | --- | --- | --- |
| mean years to discharge | **<.0001** | **<.0001** | **<.0001** |
| Mean # of assessments per 12 months time | **<.0001** | **<.0001** | **0.0052** |
| Home support hrs per week, at baseline | **<.0001** | **<.0001** | **0.0002** |
| Home support hrs per week, last 90 days observed | **<.0001** | **<.0001** | **<.0001** |
| % increase baseline to end | **<.0001** | **<.0001** | **<.0001** |
| Any nursing, first 90 days | **<.0001** | **<.0001** | **<.0001** |
| mean total hours (among those with any) | **0.0017** | **<.0001** | **<.0001** |
| Proportion of these hours by RN | **0.0093** | 0.3285 | **<.0001** |
| Any nursing, last 90 days | **<.0001** | **<.0001** | **<.0001** |
| mean total hours (among those with any) | **0.0218** | **<.0001** | **<.0001** |
| Proportion of these hours by RN | **<.0001** | **<.0001** | **<.0001** |
