# Supplementary material for: Inter-provincial variation in older home care clients and their pathways: a population-based retrospective cohort study in Canada
Source: BMC Geriatr. 2023 Jun 26;23:389. doi: 10.1186/s12877-023-04097-5 (PMC10291815; doi:10.1186/s12877-023-04097-5)
Supplement: Supplementary file 3 — Additional file 3. Statistical Difference p values for Descriptive Characteristics by Province and Discharge Pathway: Last assessment compared to Initial Assessments (Reference Table 3). [file 12877_2023_4097_MOESM3_ESM.docx]

Additional file 3: Statistical Difference p values for Descriptive Characteristics by Province and Discharge Pathway: Last assessment compared to Initial Assessments (Reference Table 1)

|  | **WRHA** | | | | | **Nova Scotia** | | | | |
| --- | --- | --- | --- | --- | --- | --- | --- | --- | --- | --- |
|  | died | LTC | still on service after 4 years | all other discharges | **All** | died | LTC | still on service after 4 years | all other discharges | **All** |
| co-resides with primary or secondary | 0.2243 | **0.0015** | **0.0002** | **0.0109** | **<.0001** | 0.4979 | **<.0001** | **0.0416** | 0.9567 | **<.0001** |
| no informal caregiver | 0.6681 | 0.7376 | 1.0000 | 1.0000 | 1.0000 | 0.6070 | 0.8105 | **0.0431** | 0.8580 | 0.0747 |
| caregiver distress | 0.6218 | **<.0001** | **0.0146** | 0.2398 | **0.0001** | **0.0009** | **<.0001** | 0.4116 | 0.5652 | **<.0001** |
| primary caregiver child/child-in-law | 0.2811 | 0.1688 | **0.0038** | 0.2886 | **0.0007** | 0.6810 | 0.0940 | 0.9160 | 0.8360 | 0.1430 |
| primary caregiver spouse | 0.1971 | 0.1729 | **0.0010** | 0.1794 | **0.0002** | 0.2115 | **0.0040** | **0.0034** | 0.3859 | **<.0001** |
| inf hours in 7 days | **0.0035** | **<.0001** | 0.0972 | 0.3530 | **<.0001** | **0.0120** | **<.0001** | 0.3589 | 0.7401 | **0.0008** |
| ADL hierarchy 1 or greater | **<.0001** | **<.0001** | **<.0001** | **0.0473** | **<.0001** | **<.0001** | **<.0001** | **<.0001** | **0.0471** | **<.0001** |
| CPS 3 or greater | **<.0001** | **<.0001** | **<.0001** | **0.0009** | **<.0001** | **<.0001** | **<.0001** | **<.0001** | **0.0002** | **<.0001** |
| CHESS 2+ | 0.0678 | **<.0001** | **<.0001** | **0.0001** | 0.2490 | **0.0091** | **<.0001** | **<.0001** | 0.2744 | **0.0001** |
| DRS 3+ | 0.1270 | **<.0001** | 0.1137 | 0.8285 | **<.0001** | **<.0001** | **<.0001** | 0.0919 | 0.1786 | **<.0001** |
| daily pain | 0.5888 | 0.5827 | **0.0004** | 0.3179 | 0.0561 | 0.2668 | 0.7138 | 0.2997 | 0.2003 | 0.4718 |
| MAPLe high or very high | **<.0001** | **<.0001** | **<.0001** | **0.0351** | **<.0001** | **<.0001** | **<.0001** | **<.0001** | **0.0010** | **<.0001** |
| urinary incontinence at least 2x per week | **<.0001** | **<.0001** | **<.0001** | **0.0079** | **<.0001** | **<.0001** | **<.0001** | **<.0001** | **0.0294** | **<.0001** |
| any aggressive behaviour | **<.0001** | **<.0001** | **<.0001** | **0.0394** | **<.0001** | **<.0001** | **<.0001** | **<.0001** | **0.0037** | **<.0001** |
| 1 or more falls | 0.0726 | 0.2966 | **<.0001** | **0.0080** | **<.0001** | 0.1352 | 0.7444 | **<.0001** | 0.0607 | **0.0001** |
| 4 or more diagnoses | **<.0001** | **<.0001** | **<.0001** | **<.0001** | **<.0001** | **<.0001** | **<.0001** | **<.0001** | **0.0001** | **<.0001** |
| Alzheimer’s/related dementia | **0.0001** | **<.0001** | **<.0001** | **0.0001** | **<.0001** | **<.0001** | **<.0001** | **<.0001** | **0.0363** | **<.0001** |
| stroke | **0.0526** | **0.0012** | **0.0028** | 0.3788 | **<.0001** | **0.0339** | **<.0001** | **0.0322** | 0.2593 | **<.0001** |
| heart failure | **<.0001** | **0.0001** | **<.0001** | 0.2011 | **<.0001** | **0.0007** | **0.0006** | **0.0326** | 0.1660 | **<.0001** |
| cancer | **0.0021** | 0.0810 | 0.1719 | 0.2568 | **0.0002** | **0.0012** | **0.0121** | 0.4425 | 0.1870 | **<.0001** |
| psychiatric diagnosis | 0.0886 | **<.0001** | **0.0001** | **0.0429** | **<.0001** | **0.0035** | **<.0001** | **<.0001** | 0.1185 | **<.0001** |
| COPD | **0.0113** | **0.0069** | **0.0002** | 0.1491 | **<.0001** | **0.0251** | **<.0001** | **0.0020** | 0.1690 | **<.0001** |
| diabetes | 0.4772 | 0.1836 | 0.1222 | 0.2977 | **0.0190** | 0.2394 | 0.2111 | 0.5474 | 0.1030 | **0.0374** |
| arthritis | **0.0053** | **<.0001** | **<.0001** | **0.0398** | **<.0001** | **0.0040** | **<.0001** | **<.0001** | **0.0447** | **<.0001** |
